# Real-Time Wood Behaviour: The Use of Strain Gauges for Preventive Conservation Applications

**DOI:** 10.3390/s20010305

**Published:** 2020-01-06

**Authors:** Willemien Anaf, Ana Cabal, Mie Robbe, Olivier Schalm

**Affiliations:** 1 ARCHES, Conservation Studies, University of Antwerp, Blindestraat 9, B-2000 Antwerp, Belgium; willemien.anaf@uantwerpen.be (W.A.); mieke.robbe@uantwerpen.be (M.R.); 2 Department of Physics, CEADEN, 502, Calle 30, Havana 11300, Cuba; ana.cabal@uantwerpen.be; 3 Antwerp Maritime Academy, Noordkasteel Oost 6, B-2030 Antwerp, Belgium

**Keywords:** wood, strain gauges, monitoring, heritage, preventive conservation

## Abstract

Within the heritage field, the application of strain gauges on wood surfaces is a little-explored but inexpensive and effective method to analyse the environmental appropriateness of rooms for the wooden heritage collections they contain. This contribution proposes a wood sensor connected to a data logger to identify short moments with an elevated risk of harm. Two experiments were performed to obtain insights pertaining to the applicability of wood sensors to evaluate preservation conditions. (1) The representativeness of strain gauges on dummies was tested for their use in evaluating the preservation conditions of a range of wooden objects exposed to the same environment. For this, three situations were mimicked: a bare wood surface, a wood surface covered with a preparation layer, and a wood surface covered with a preparation and varnish layer. (2) The usability of strain gauges to monitor the wood behaviour in real-time measurements was tested with a monitoring campaign of almost two years in a church where a new heating system was installed. The results of both experiments are promising, and the authors encourage a broader application of strain gauges in the heritage field.

## 1. Introduction

A common practice in the heritage community is to extend the lifetime of wood, which is an omnipresent material in museums, churches and depots, by improving the overall preservation conditions. The environmental appropriateness of a room is usually evaluated by comparing the measurements of only a few parameters (i.e., temperature (T) and relative humidity (RH)) obtained from a limited number of points with a variety of standards. Unfortunately, the assessments based on these standards do not necessarily result in consistent outcomes. However, all standards rely on the same principles: (1) RH must remain as stable as possible, and (2) T fluctuations are less harmful than RH-fluctuations, but must also fulfil specific boundary conditions [1]. All standards acknowledge that hygroscopic wood absorbs or desorbs moisture from the surrounding environment until it reaches a new equilibrium with RH and T. This moisture uptake and release is accompanied with dimensional changes in the wood (i.e., strain). Due to the anisotropic character of wood [1,2,3] and the different behaviour rate between surface and core [2,4,5], the strain is not homogeneous throughout the material and this results in stress. Stresses in wood are an important hazard for damage, causing fracture, permanent deformation, etc. Restrained wood (i.e., wood that cannot freely move, such as a panel painting inside a frame) undergoes an additional external force. Unfortunately, indoor monitoring campaigns in heritage sites have demonstrated the difficulty to keep constant relative humidity conditions [6,7,8,9]. Due to varying environmental humidity conditions, wooden objects must continuously adapt themselves. The currently used monitoring method uses indirect information (i.e., T and RH) to determine whether the dimensional changes of wood are acceptable.

The variability of indoor environmental conditions results in moments of appropriate preservation conditions interspersed with periods of higher probability whereby wood is damaged. Instead of improving the overall preservation conditions, the lifetime of wood can also be extended by implementing dedicated mitigation actions that avoid the recurrence of small periods of elevated risk in the future. However, with only T and RH measurements it is not always obvious to identify such periods. Direct information on the continuous wood behaviour could represent an important additional information source to improve the identification of periods with elevated risk. Several existing methods can be used for that purpose:

- Mathematical relations can be used to determine the equilibrium moisture content in wood and the accompanying dimensional change from the acquired T and RH measurements. This means that environmental methods can be post-processed to optimize the environmental preservation conditions [10]. However, in practice, it remains complex to estimate how wooden artefacts respond to their environment. The complexity is even aggravated when the wooden surfaces are treated with oils, waxes, lacquers, or varnishes, or in the case of painted wood, with preparation, paint, or varnish layers. Such layers affect the moisture exchange between the object and environment. When not all of an object’s surfaces are covered with a coating, an additional problem of uneven dimensional response can occur, inducing additional stress [11].
- Several non-contact techniques are used to measure the deformation of the complete (painted) surface of a genuine object. Examples of such techniques are digital speckle pattern interferometry (DSPI) and speckle decorrelation (DIC) [12,13,14,15]. Structured light-based scanners are also explored [16].
- An alternative approach is to directly monitor the dimensional changes of wood using sensors. A well-known method is the deformometric kit (DK) that is developed to monitor the deformation of panel paintings [17,18]. The DK measures both the panel’s swelling and shrinking behaviour as its distortion (cupping). The DK is mounted at the backside of the panel painting and monitors the behaviour of the panel as a whole. The same research group also developed a monitoring cross beam (MCB) that measures cupping, swelling and shrinking, and constraint forces [19]. Strain measurements on wood are also performed with fibre Bragg grating sensors [13,20,21]. Another technique makes use of strain gauges that are glued on the wood surface [13,22,23,24,25,26]. Most of the techniques mentioned are invasive because they require screws or glue. For ethical considerations, heritage caretakers forbid the applications of such techniques on genuine objects.
- Direct monitoring of stress damage using a sensor that measures acoustic emissions gives a direct insight into the evolution of accumulated damage [27,28,29].

The idea of this study is to analyse the feasibility of assessing indoor conditions by directly monitoring material behaviour. Since the heritage community does not allow the application of strain gauges on genuine objects of art, a small wooden block to which a strain gauge has been applied is placed in the room where the objects of art are housed. Data obtained from this wood sensor are used to assess the environmental conditions of that room. This method has the advantage that it is relatively inexpensive, non-invasive and moveable. In addition, it does not require high end expertise, as is needed for some of the previously mentioned methods. However, before this sensor is accepted by the heritage community, the following questions must be answered:

- Can the collected information about the real-time shrinkage and expansion of a dummy block of wood be generalized to other wooden heritage objects stored in the same room? In one single room, there could be a large variation of wooden objects, all exposed to the same environmental conditions. Apart from different types of wood, thicknesses, and variations in construction, the objects could also have a wide variety of finishing layers (varnish, oil, wax, lacquer, paint, etc.). Experiments are performed in laboratory conditions to test the impact of a preparation and varnish layer on the swelling and shrinking behaviour of a wooden support.
- Is the real-time monitoring of wood behaviour usable to evaluate the environmental appropriateness? A measuring campaign of almost two years was performed in a church where a new heating system was installed. In this real-life condition, a wood sensor was used to gather information on the influence of the heating system on the wooden church interior, and to gain better insights into the appropriateness of the environmental conditions. In addition, a method is proposed that facilitates the interpretation of the time series: the behaviour is decomposed in a behaviour in the low frequency, mid-frequency, and high-frequency range.

## 2. Materials and Methods

### 2.1. Strain Gauges

**Figure 1:**
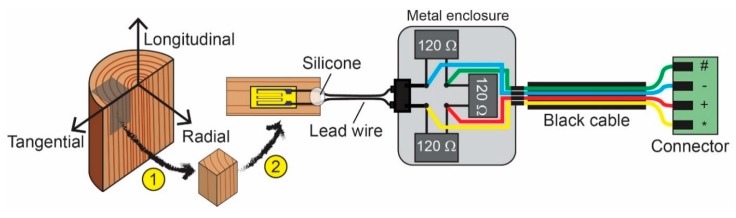
Schematic diagram of the wood sensor and electronic circuit. In step 1, a smaller block of wood is cut from a tree trunk. In step 2, the surface is prepared to attach a strain gauge on the surface along one of the three anisotropic directions of the wood. Then, the strain gage is coupled to the completion circuit to form a Wheatstone bridge inside a metal enclosure. A four-wired cable connects the Wheatstone bridge with the datalogger.

The construction of the proposed wood sensor is shown in Figure 1. It makes use of a strain gauge. Strain gauges are relatively inexpensive (around 30 euro for one gauge). A strain gauge converts the mechanical material strain due to changing hygro-thermal conditions into a change in electrical resistance. The strain gauge responds to the average strain conditions under its measuring grid. To average out the strain distribution in heterogeneous materials such as wood, a gauge length of 4 to 5 times the heterogeneities is required. Therefore, the PFLW-30-11 strain gauge (Tokyo Measuring Instruments Laboratory, TML, Tokyo, Japan) with a length of 30 mm and dedicated for wood, has been selected. A metal foil lined at the back of the strain gauge protects it from the influence of moisture in the wood. The used strain gauges have a nominal resistance *R*_0_ of 120.3 ± 0.5 Ohm, a gauge factor *K* of 2.13, and 2 pre-attached extension lead wires of 1 m. The strain limit of the gauge is 20,000 ppm. As recommended by the TML-company, the gauges were bonded to the wood surface using the PS adhesive, a two-component room-temperature-curing polyester adhesive. After a thorough surface cleaning, the typical procedure for strain gauge adherence was followed.

The strain gauge was included in a completion circuit to form a Wheatstone bridge. This enabled the measurement of small changes in resistance. The 3 completion resistors had the same resistance as the nominal gauge resistance *R*_0_ (high precision foil resistors of 120 Ohm, S-series, Vishay Precision Group, Malvern, PA, USA). The resistors had a temperature coefficient of resistance of about 2 ppm/°C (i.e., the change in resistance as a function of the ambient temperature). The completion circuit was built inside a metal enclosure (Hammond Manufacturing, Guelph, Ontario, Canada).

The change in resistance, Δ*R*, was monitored over time by connecting the Wheatstone bridge to a datalogger (DataTaker DT85, Thermo Fischer Scientific, Scoresby, Australia). The quarter bridge arrangement was excited with a constant current of 2.5 mA through 2 power lines connected to opposite corners of the bridge (i.e., a current bridge). Although less accurate than a voltage bridge, a current bridge has a lower noise susceptibility. The excitation voltage *V_ex_* was calculated based on the known arm resistances of 120 Ohm. The output voltage *V_out_* of the bridge was determined across the other corners.

The measurement output of the bridge as measured by the DataTaker is expressed as a ratiometric form *B_out_* = *V_out_/V_ex_* in parts per million (ppm). For a quarter bridge arrangement, the relation between *B_out_* and the relative change of resistance Δ*R*/*R*_0_ is known. The gauge factor *K* describes the relation between the relative change in resistance and the relative change of length of the wood Δ*L*/*L*_0_, expressed as micro strain (i.e., strain × 10^6^). A 1 ppm expansion should be interpreted as a wooden beam of 1 m that expands with 1 µm.

$$B_{out}=\frac{V_{out}\left[V\right]}{V_{ex}\;\left[V\right]}\times 10^{6}=\frac{1}{4}\frac{\Delta R\;[\Omega ]}{R_{0}\;[\Omega ]}\times 10^{6}=\frac{1}{4}\left(K\times \mu \varepsilon \;\left[µm/m\right]\right)\;\overset{}{\Leftrightarrow }\mu \varepsilon \;\left[µm/m\right]=\frac{4}{K}B_{out}$$

By subtracting the first measurement from of all the subsequent measurements of *B_out_*, the dimensional changes relative to the initial situation are obtained. This procedure also compensates for the offset. The same setup as Figure 1, but with a strain gauge not adhered to any substrate and kept flat underneath a weight, was used to measure the impact of fluctuating indoor T and RH on the readout of *B_out_*. The standard deviation is 18.38 ppm for a measuring period of 23 days, suggesting a small effect on the measured strain. During that experiment, the wood sensor operated at the same time. The wood sensor described in paragraph 2.3 fluctuated between −2944 ppm (i.e., minimum value) and 4097 ppm (i.e., maximum value). This means that the signal of the wood sensor is below the strain limit and clearly higher than the background signal generated by a freely moving strain gauge. The sensitivity of the strain gauge is described by the gauge factor and certified by the company TML.

### 2.2. Feasibility Study

Short-term experiments were performed to study whether the effect of finishing layers on the wood swelling and shrinking behaviour could be sensed by the strain gauge. Six rectangular pieces of quarter sawn oak (4.5 × 10 cm) were used to prepare the wood sensors. Three of the pieces had a thickness of 3 mm, the other three were 6 mm thick. On the surface of each wooden pane, a strain gauge was applied on the longitudinal direction and connected to the datalogger as described in the previous paragraph. The longitudinal direction is the direction with the least dimensional change. If the effect of finishing layers is visible in this orientation, then this can also be observed in the other orientations. Environmental temperature (T) and relative humidity (RH) conditions close to the wooden panes were measured by connecting a calibrated Telaire T9602 T and RH sensor (Amphenol, St.-Marys, PA, USA) to the same datalogger. The signal of all sensors was captured simultaneously with a time interval of 1 min.

**Figure 2:**
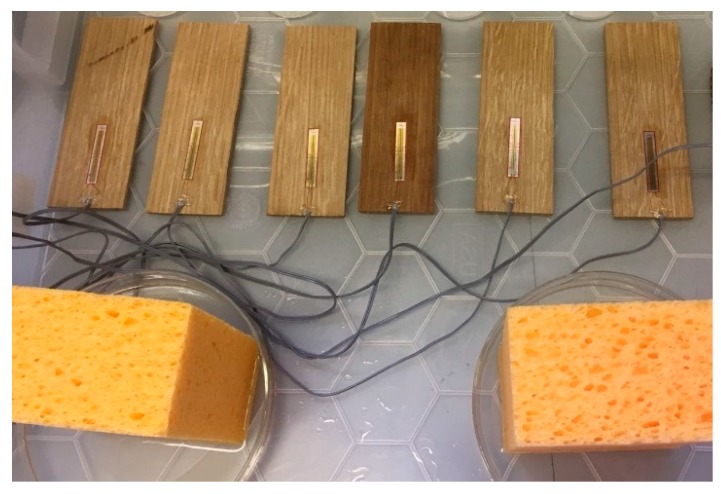
Picture of the experimental setup. Six wood panes with strain gauges adhered. Petri dishes with sponges soaked in water are used as humidity source.

All experiments were performed inside a plastic box in which step changes in RH have been generated (see Figure 2). The datalogger was kept outside the box. The connection wires between sensors and logger were led through a small opening in the box which was covered with tape to minimize the air exchange rate. An open box generated intermediate RH levels, corresponding to ambient indoor conditions. An RH step increase was induced by adding a humidity source (petri dishes with sponges soaked in water) and closing the box. An RH step decrease was generated by opening the box and removing the humidity source. Three subsequent experiments were performed.

1. Untreated wood: The surface behaviour of 6 untreated wood sensors were monitored while exposed to the same step changes in RH.
2. Preparation layer: After the experiment with the untreated wood, 4 wood sensors (2 thin and 2 thick ones) were covered with a chalk-glue preparation layer. The strain gauge glued on the wood surface was covered by this preparation layer. Such a preparation layer is representative of the 14th–17th century tradition north of the Alps. This was prepared by dissolving 10 g rabbit-skin glue in 100 mL of water. The liquid mixture was heated in a water bath, and chalk was added until the saturation point was reached (around 90–100 g of chalk). First, two layers of warm glue were applied with a bristle spalter to fill all wood pores and to cover the strain gauges in order to obtain a good adhesion of the preparation layer. Subsequently, two layers of the chalk–glue mixture were applied with a filler spatula to obtain a uniform layer. The four lateral edges were prepared analogously. After drying, the surface of the preparation layer was slightly sanded with 200 grit sandpaper. All wood sensors were again monitored while exposed to the same RH step changes. The two untreated wood sensors were considered as a reference.
3. Varnish layer: The 4 wood sensors with a preparation layer were covered with a thick dammar-based varnish layer. The dammar was dissolved in white spirit (30 g in 100 mL). To avoid edge effects, the varnish was applied on the four lateral edges as well. Subsequently, all wood sensors were simultaneously exposed to RH step changes, with the two untreated sensors as reference.

### 2.3. Practical Application

A long-term measurement campaign of 21 months was performed to evaluate the usability and added-value of the wood sensor in real-life situations. An organ loft (7 m height) in a late Gothic church in the centre of a small Belgian city served as the measuring location. The church houses several wooden statues, decorative elements, and a pulpit.

The used wood sensor consisted of a strain gauge glued in the radial direction on block of 16^th^ century oak (10 × 10 × 5 cm). The wood originates from the beam of an historic house and was dated with dendrochronology. The beam was not in its original position anymore but kept in the basement of a historic house. A small piece was sacrificed to be used as a sensor. Due to the curvature of the wood rings and the rather long strain gauges, it was hard to isolate the tangential orientation (i.e., orientation with the highest dimensional change) from the other ones. The radial direction was selected because of practical issues and because it exhibits an average behaviour. Complementary to the wood behaviour, temperature and relative humidity data were collected with a GMW90 (Vaisala, Helsinki, Finland). Both sensors were connected to a DataTaker DT85 and were read out in phase with a time interval of 15 min. Monitoring started on 3 July 2017 and continued until 21 March 2019. From June to December 2017, a new heating system was installed in the church, based on convection heating at low temperatures. The target temperature of the heating is set at 10 °C. When outdoor temperatures increase during the warmer seasons, the heating system is automatically switched off. The system was first started-up on 25 January 2018, heating the church until 1 April 2018. In the next winter period, the heating system was switched on from 28 October 2018 until 17 February 2019, and from 3 March 2019 until the end of the measuring period.

In addition to the monitoring of the wood behaviour in the church, we also propose a method for processing these measurements. The collected data streams of RH and strain are split in 3 frequency ranges using a method that is described elsewhere [6]. Some standards and norms for the preservation of wood propose different recommendations for seasonal and other fluctuations. This method facilitates the comparison between measurements and such recommendations. In addition, the method allows the isolation of the swelling and shrinking caused by several hazards:

1. Seasonal fluctuations: Low-frequency fluctuations occur at time scales of several months. Such fluctuations are determined by calculating a central 30-day moving average on the raw data stream.
2. Weather changes and other medium frequency variations: These fluctuations occur at a time scale of several days to weeks, and they are isolated by subtracting the 30-day moving average from the raw data, and subsequently suppressing the high-frequency fluctuations by using a central 24-h moving average.
3. Day–night and other fast-occurring fluctuations: The high-frequency fluctuations occur in a time-scale of hours and are determined by subtracting the low- and mid-frequency fluctuations from the raw data.

## 3. Results

### 3.1. Feasibility Study

**Figure 3:**
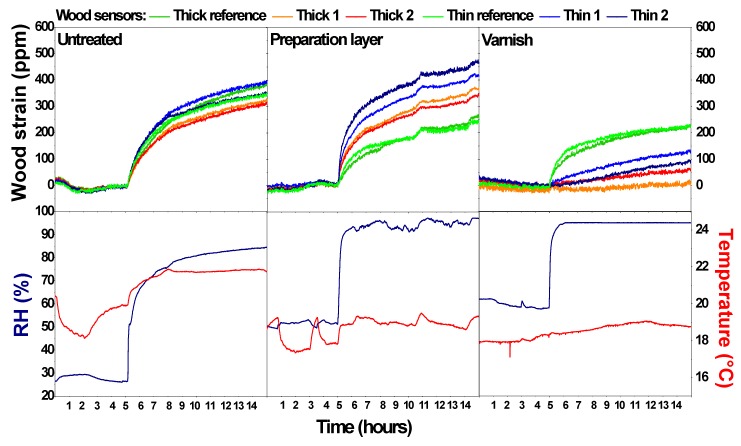
Dimensional response of the wood sensors for an RH step increase generated in a closed box in three different situations: (1) untreated wood, (2) wood with a preparation layer, and (3) wood with a preparation and varnish layer. The corresponding temperature and relative humidity conditions are shown for each situation.

**Figure 4:**
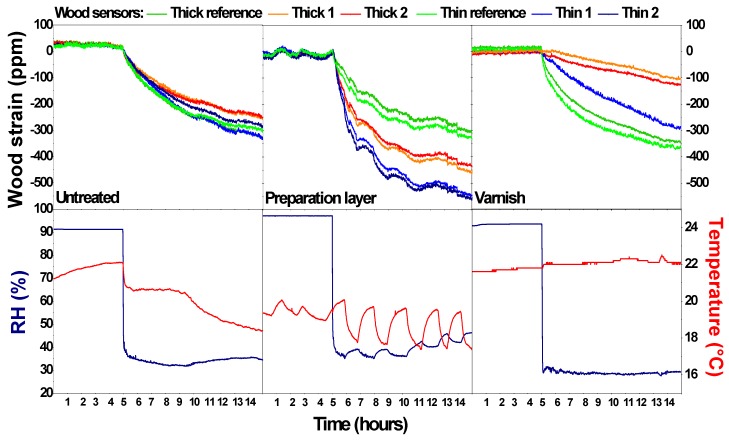
Dimensional response of the wood sensors for an RH step decrease generated in a closed box in three different situations: (1) untreated wood, (2) wood with a preparation layer, and (3) wood with a preparation and varnish layer. The corresponding temperature and relative humidity conditions are shown for each situation.

Figure 3 gives an overview of the surface behaviour of the untreated wood, the wood with preparation layer, and the wood with preparation and varnish layer for an RH step increase. The corresponding temperature and humidity conditions are plotted below each situation. Figure 4 gives an overview of the wood sensors behaviour for an RH step decrease. The figures show the period 5 h before up to 10 h after the RH step change. The RH steps generated inside the closed boxes are all about 40–60%, although a superposition of some smaller fluctuations due to changing indoor conditions can be seen as well. The contribution of both types of environmental change to the dimensional change is sufficiently large to isolate the impact of the RH steps. The strain gauge data were processed, considering the data point at the moment of the RH change as the reference with a ppm value of zero. All other values were recalculated accordingly.

In experiment 1, the six untreated wood surfaces behave in a similar way for both the RH increase and decrease. The small differences could be due to differences between the wood sensors (difference in heterogeneity, none of the strain gauges is adhered on a surface that is perfectly longitudinal, etc.). This means that the six wood sensors detect the same inappropriate moments. The sudden change in RH results in a more progressive behaviour of the wood surface. Within the first hour, the wood surface changes fast but over time the change becomes smaller. An RH-change of around 55–60% that occurred in a matter of minutes results in a wood swelling or shrinking of around 300–350 ppm in 10 h. In the tested time-frame, the swelling and shrinking behaviour of the wood surface is not affected by the thickness of the panes, although the total shrinkage and swelling after 10 h might be slightly higher for the thin panes. The differences between R and wood behaviour trends indicate that the wood sensor is able to deliver complementary information to classic monitoring campaigns solely based on T and RH.

In experiment 2, two wood sensors remained untreated while the other 4 were covered with a preparation layer. Both the thick and the thin (untreated) reference sensors behave in a similar way with a similar dimensional change as in experiment 1. The sensors with the preparation layer show a higher dimensional change, for both the RH increase and decrease. Moreover, a clear distinction is present between the thin and the thick sensors, with a higher dimensional response of the thin sensors. This behaviour has also been observed in other RH step increases (not shown here). This higher dimensional response can be explained by the hygroscopic nature of the preparation layer: chalk and certainly the glue attract and hold moisture at the surface. With only this effect, a similar strain would be expected for the thin and thick wood sensors. Therefore, an additional effect must be present, probably a cupping effect (warping) enhancing the strain as measured by the strain gauge [1,11,25]. The combination of the expansion and cupping results in a higher dimensional change at the wood surface below the preparation layer (Figure 4). This effect is greater for thinner pieces of wood. This means that some finishing layers can reinforce the wood behaviour and this should be considered when information of the wood sensor is extrapolated to a large heritage collection. Also, the thickness of the wood panel affects the behaviour.

**Figure 5:**
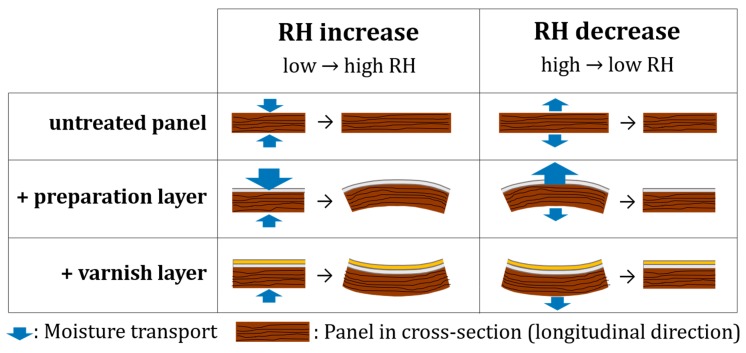
Schematic overview of the dimensional response of quarter sawn panels (see Figure 2 for the setup). The cross-sections in the longitudinal direction visualize the response for untreated wood, wood with preparation layer and wood with preparation layer and varnish, for increasing and decreasing RH conditions. The moisture transport at the preparation layer is enhanced compared to the opposite untreated side, while this transport at the surface covered with a varnish layer is inhibited compared with the untreated opposite side.

In experiment 3 (varnish layer on top of preparation layer), the RH step change is comparable to that of experiment 1 (untreated wood) and 2 (with preparation layer). The two reference sensors show a similar dimensional response as experiments 1 and 2 for both thicknesses, with a dimensional change of around 250–350 ppm. For the wood sensors with a varnish layer, the dimensional response is strongly reduced. This reduction is the highest for the thick wood sensors (variations between hardly any dimensional response to around 100 ppm). The varnish acts as a water vapour barrier, highly decreasing the water vapour flux [30]. The limited response still present could be due to a cupping effect reducing the measured strain (Figure 5), defects present in the varnish layer, or moisture entering from the (untreated) back of the sensor. This means that other finishing layers minimize the wood behaviour. However, the presence of varnish layers on for example panel paintings should not be used as an argument that dimensional changes would be minimal, because once this barrier fails, this layer can reinforce dimensional changes. Moreover, an unequal water vapour barrier at front- and backside might be detrimental. In these experiments, the thickness of the wood panels affected the wood behaviour.

The feasibility study shows that six untreated wood samples respond simultaneously to a sudden RH change, although the wood requires time to achieve a new equilibrium with its environment. This suggests that all wood objects in a varied heritage collection will respond at the same time to the same environment. Untreated wood can be considered as an estimator of the average behaviour of the wooden collection. Inappropriate moments resulting in sudden dimensional changes can directly be observed with the wood sensor, and this information facilitates the interpretation of T and RH measurements.

The wood sensor also provides information about the level of risk that a wooden object endures. The level of risk is related to the extent of dimensional change. The experiments have shown that the extent of dimensional change is affected by several parameters, such as the finishing layer and the wood thickness. The preparation layer enhances the level of risk while the varnish layer reduces the level of risk. This means that a varied wood collection will endure dimensional changes in different degrees.

### 3.2. Practical Application

**Figure 6:**
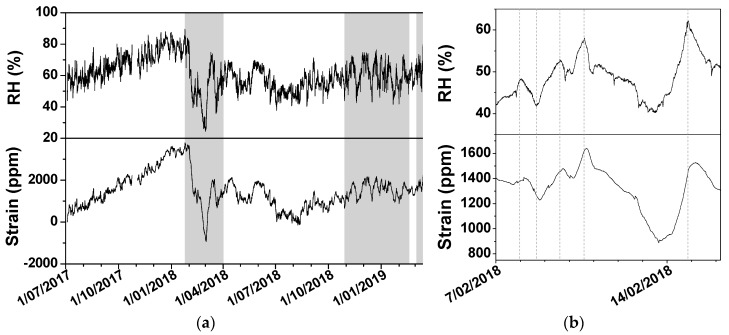
(**a**) relative humidity and wood strain in the period 3 July 2018 inside a Belgian church. The periods with heating are marked in grey. (**b**) detail for the period 3 July 2018. Visual indication with dashed lines to demonstrate variable shift between RH and strain data.

Figure 6a shows the data of the relative humidity and wood strain in a church over a period of 21 months. The grey areas are the periods the heating system was switched on. The monitoring campaign demonstrates the variability of the indoor environment and the reason why the preservation conditions must be assessed for every moment in time. The wood behaviour follows the trend in relative humidity, although high-frequency fluctuations in RH are remarkably less reflected in the strain. The increasing trend that is visible until the end of January is abruptly countered when the heating system started up. Afterwards, several large fluctuations appear. In about one month, the wood sensor records a shrinkage of around 4500 ppm. This exceeds the commonly considered average yield point for non-degraded wood, which is 0.4%, corresponding to 4000 ppm [31,32]. Above this yield point, plastic deformation can occur. However, this yield strain is conservative: Strain hardening can significantly increase the yield strain. Moreover, the time-dependent mechanical properties of wood are not considered, such as fatigue limit, creep and relaxation [33]. In addition, wood can withstand larger RH-fluctuations at lower temperatures because such conditions lower the moisture diffusion coefficient in wood [11]. After one year, the strain in the wood is again around its initial level. We assume that the behaviour of the wood inside the church follows a similar trend as the dummy block of wood, although the extent of dimensional change can be different. The feasibility study has demonstrated that not all finishing layers have protective properties. Therefore, one cannot assume that objects are protected against the hazard of fluctuating preservation conditions. For restrained wood, the level of risk will show a similar trend as unrestrained wood, not because of the internal stress, but because of the presence of external forces.

The experiments in the feasibility study showed that the wooden boards responded almost immediately to RH changes but that the evolution towards the equilibrium requires more time. The zoom in Figure 6b of a period of 10 days suggests a time shift between the RH trend and the trend in the strain data. That time shift is most probably the result of the slower evolution towards a new equilibrium. The shift between both trends is more obvious for the sharper peaks and drops and less pronounced for the low frequency fluctuation.

**Figure 7:**
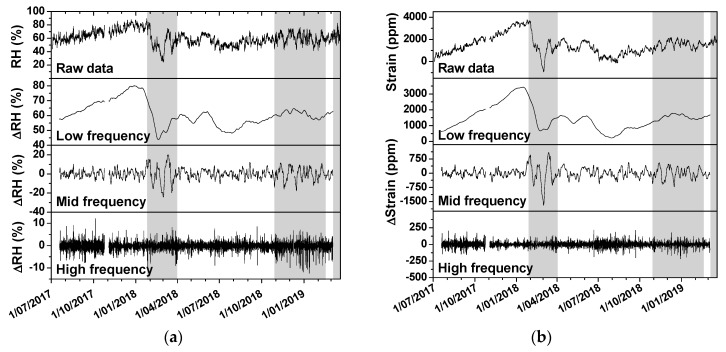
Data of relative humidity (**a**) and wood behaviour (**b**), with isolation of low, mid and high frequencies.

Fluctuations in relative humidity are considered as an important key risk indicator for damage in hygroscopic materials such as wood [34]. Figure 7 shows the presence of fluctuations in different frequency ranges. Each range has a different impact on wood and can for that reason be associated to a different level of risk. Therefore, a method is proposed that allows a fluctuation analysis, isolating fluctuations in three different frequency ranges: low, mid and high. The raw data of RH and strain were processed according to this method. In the mid-frequency range, a significantly different behaviour can be noticed in both the RH and strain data between the periods with and without heating. In the periods with heating, a higher amplitude can be observed, and the peaks are broader. This behaviour is strongly related to the outside temperature affecting the indoor environment. In cold periods, cold outdoor air is heated by the system and introduced in the church. The colder the outdoor air, the more the air must be heated and the lower the relative humidity becomes. During the start-up of the heating system, the security system that stops the heating when the RH undershoot a threshold was not yet activated.

**Figure 8:**
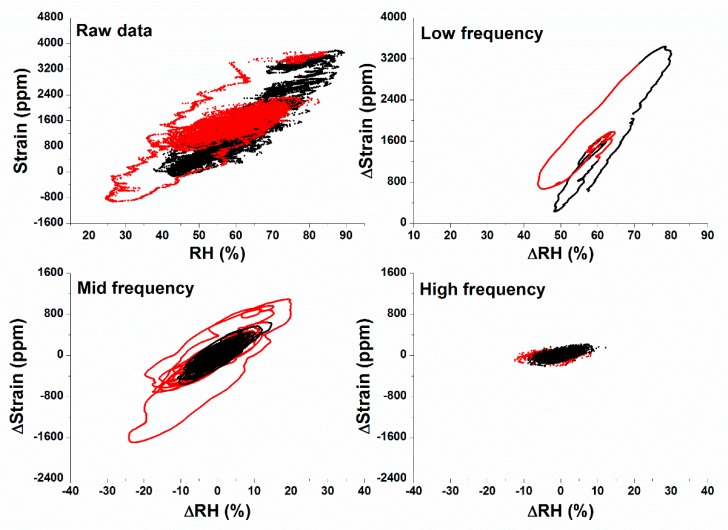
Strain versus relative humidity for the raw data, and for three different frequency domains. Data points when the heating system was switched on, are in red. The graphs of the three frequencies have the same window size: ΔRH = 80% and ΔStrain = 4000 ppm.

To get more in-depth information on the RH-strain relation, both parameters were plotted against each other for the raw data and the three frequency ranges. Figure 8 shows the four resulting scatter plots. Data collected in periods with the heating system switched on are marked in red. The strain axes of the three graphs with isolated frequency domains cover a range of 4000 ppm. Notably, in contrast to the raw data, all isolated fluctuation data stay within this 4000-ppm window. The linear regression slope estimates the hygro-mechanical reactivity, with a higher slope indicating a higher reactivity [8]. The slopes calculated for the raw data, and the low, mid and high frequency fluctuations, are respectively: 71, 85, 45, and 10 (not shown in the graphs). Thus, RH-fluctuations in the low-frequency range result in the highest dimensional response. This is due to the fact that the wood (fully) responds to each RH-cycle.

- Low frequency range: The low frequency scatter plot shows a limited number of large cycles, mainly corresponding to seasonal changes. Although the swelling and shrinking behaviour of the wood is the largest in this frequency domain, it is expected that the changes in the wood occur slowly such that the wood can adapt to the changing internal stresses, and stresses due to differences between the surface and core are limited.
- Mid-frequency range: The RH-fluctuations in the mid-frequency range result in a significant swelling and shrinking behaviour. The duration of the fluctuations last long enough that the deeper ‘layers’ of the wood can adapt to the changing environmental conditions [35]. During the periods with heating, these cycles are characterized by higher RH-fluctuations and therefore, also higher strain values. Fluctuations in the mid-frequency fluctuations, on the contrary, show many cycles. This results in a continuous change in the wood stresses.
- High frequency range: The high frequency RH-fluctuations induce small changes in the wood surface strain. This is characterized by flat cycles. Although the RH-fluctuations only last few hours, they continuously force the superficial layers of the wood to adapt. However, the dimensional changes are limited, resulting in a smaller slope. This suggests that the wood surface is less sensitive to fast fluctuations and that fast RH fluctuations are less harmful than mid-frequency RH changes.

This real-life experiment demonstrates the usability of the wood sensor in long-term hydro-mechanical monitoring. The breakdown of the raw data of both RH and strain into low, mid, and high frequency fluctuations introduces an additional layer to the data interpretation. Since the duration of the RH-fluctuations has an influence on the damage potential, such analysis supports the evaluation of which fluctuations have the highest influence on the wood. It is expected that RH-fluctuations in both the mid and high frequency ranges can result in micro and macro cracks at the surface due to the internally created stresses in (massive blocks of) wood [32,36,37,38,39,40], although the practical application indicates a smaller impact of the high frequency RH changes. This could be especially problematic in the case of valuable finishing layers that do not (any longer) act as a water vapour flux barrier.

## 4. Conclusions and Future Work

This contribution has demonstrated that it is possible to estimate the environmental appropriateness of rooms for the preservation of heritage collections containing wood by measuring the dimensional change of a dummy block of wood using a strain gauge glued on the surface. The wood sensor directly measures the material behaviour and delivers information that is complementary to T and RH measurements. The dimensional change in the radial orientation of an untreated piece of wood can be considered as an estimator for the average behaviour of a wood collection.

The feasibility study demonstrated that, despite the presence of surface finishing layers, all wood will endure dimensional changes, even in the longitudinal direction. This study also showed that several untreated wood samples exposed to the same environment follow a similar trend in wood behaviour: There is an instantaneous response of the wood surface, but the sample requires more time to achieve a new equilibrium with its environment. The slower evolution towards its equilibrium results in a shift between the RH-trend and the trend in dimensional change. The feasibility study clearly showed that the level of risk to which a wooden object is exposed is affected by the finishing layer and the wood thickness. The extent of dimensional change within a wooden collection will be different for each object. One can assume that inappropriate periods identified with the wood sensor are also felt by a wooden collection exposed to the same environment, and that the presence of a finishing layer does not necessarily protect objects against RH fluctuations.

The concept of the wood sensor has been used to estimate the appropriateness of an environment wherein a new heating system has been switched on for the first time. The practical application demonstrated that the dimensional behaviour of wood can be monitored for a longer period. The dimensional change over time is subject to a contribution from low, mid and high frequency fluctuations and a method is proposed that allows for a separate analysis of the three frequency ranges. From this study, it became clear the mid-frequency dimensional fluctuations due to the heating system caused the highest levels of risk. The high-frequency RH fluctuations have a lower impact on wood than the mid-frequency RH fluctuations. If better insights are needed pertaining to the levels of risk, the wood sensor can be modified to mimic historical wood collections (e.g., type of wood, thickness, orientation of strain gauge, finishing layers, etc.). The feasibility study has demonstrated that it is possible to cover the strain gauge and the surrounding wood surface with surface finishing layers. This has the advantage that the wood surface just below the finishing layer can be monitored.

The experiments in the feasibility study are performed as a proof-of-concept. Future experiments could test more elaborate wood sensors by, for example, providing strain gauges in different orientations (tangential, radial and longitudinal), on the front- and backside of a wooden pane, with and without finishing layers. This could give provide information related to the internal restraint and cupping of wood panels. Strain gauges could also be useful for conservator-restorers to test several (innovative) options for finishing layers to compare their effect on the wood behaviour [25]. More in-depth data analysis could be performed, such as cross-correlation between RH and strain [41], to determine the response rates. When performing such analysis on the isolated fluctuation data, better insights could even be obtained from the response times of the wood on different time scales.

Since the attachment of the strain gauge to the wood is irreversible, it cannot be applied to genuine objects. For this reason, we have used dummy blocks of wood. However, once the method is well mastered, exceptions can be made by applying strain gauges to real objects. In this case, the gauges have to be glued at invisible areas of wooden heritage objects. Possible examples are the backside of wooden panelling in churches or historic houses. The small dimension and the limited thickness of the strain gauge allow for a wider range of real applications compared to, for example, the deformometric kit.
